# Supplementary material for: Neural stem and progenitor cells support and protect adult hippocampal function via vascular endothelial growth factor secretion
Source: bioRxiv. 2023 Apr 24:2023.04.24.537801. Preprint. [Version 1] doi: 10.1101/2023.04.24.537801 (PMC10168272; doi:10.1101/2023.04.24.537801)
Supplement: Supplement 1 [file NIHPP2023.04.24.537801v1-supplement-1.pdf]

bioRxiv preprint doi: https://doi.org/10.1101/2023.04.24.537801; this version posted April 24, 2023. The copyright holder for this preprint (which was not certified by peer review) is the author/funder, who has granted bioRxiv a license to display the preprint in perpetuity. It is made available under aCC-BY 4.0 International license.

## Supplemental Figures

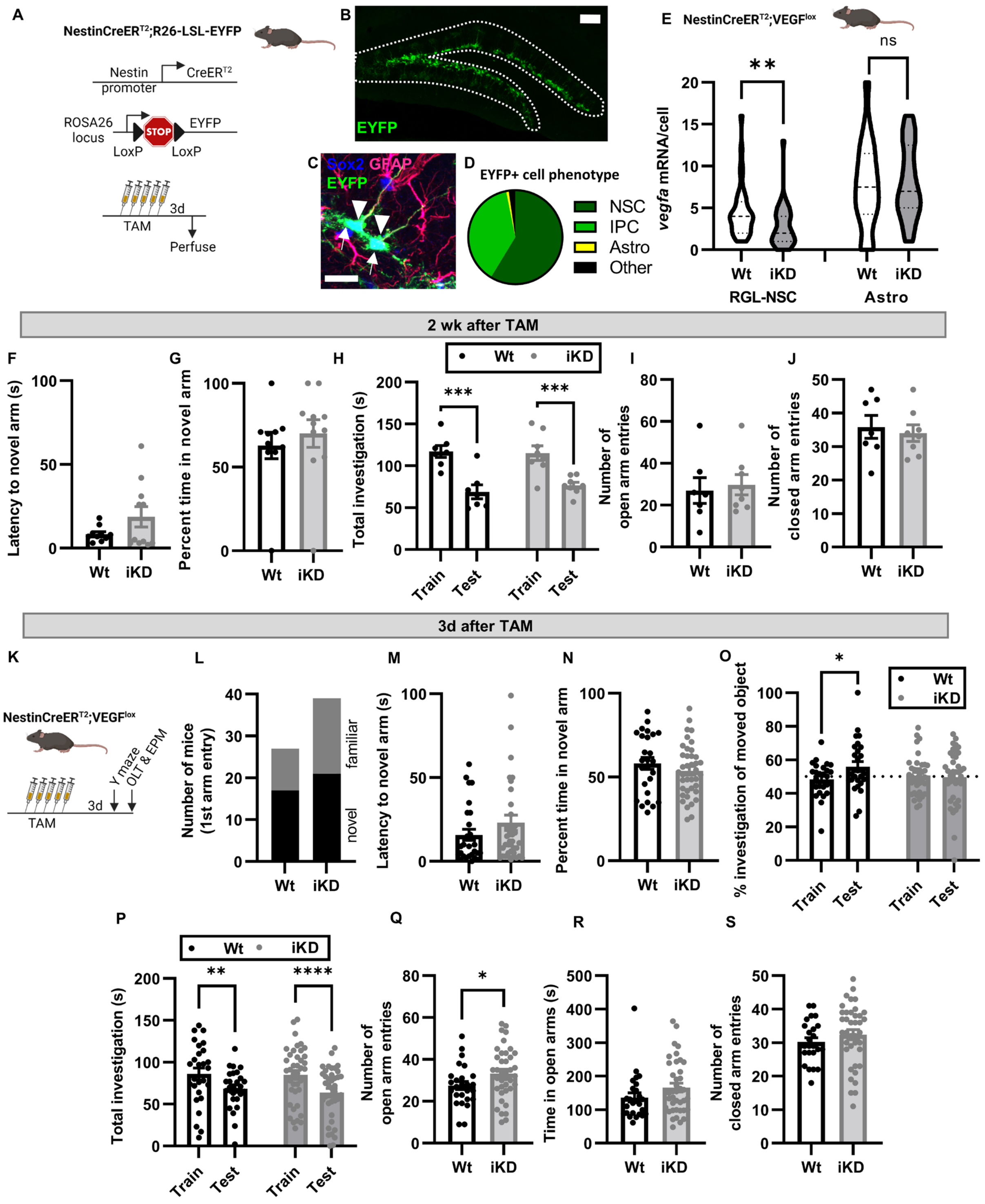


**Supplemental Figure 1. NSPC-VEGF knockdown specificity and behavioral data at 3d after TAM.** A) Schematic of transgenic mouse strains used to further assess Cre-induced recombination specificity. NestinCreER$^{T2+/-}$;Rosa-Lox-STOP-lox$^{+/-}$ mice mice were perfused 3d after TAM. B) Representative immunofluorescent images of EYFP labeling in the DG. Scale = 100 µm. C) Representative image of EYFP+ NSCs (Sox2+/GFAP+ with apical process,

bioRxiv preprint doi: https://doi.org/10.1101/2023.04.24.537801; this version posted April 24, 2023. The copyright holder for this preprint (which was not certified by peer review) is the author/funder, who has granted bioRxiv a license to display the preprint in perpetuity. It is made available under aCC-BY 4.0 International license.

arrowhead) and IPCs (Sox2+/GFAP-, arrows). Scale = 20 µm. D) Pie graph showing percent of EYFP+ cells in the DG that where phenotypic NSCs, IPCs, astrocytes or other. N = 3 mice. E) Violin plots of *vegfa* mRNA puncta per cell in Wt and iKD NSCs versus astrocytes. **p = 0.0025 Mann Whitney test. N = 40 RGL-NSCs/group, 20 astrocytes/group. F) Latency of mice to enter novel arm in Y maze. T-test, ns. N = 10 Wt, 11 iKD mice. G) Percent time in novel arm over total time in novel and familiar arm. T-test, ns. Mean ± SEM and individual mice shown. N = 10 Wt, 11 iKD. H) Total object investigation time in the object location training and testing sessions. 2-way ANOVA trial p <0.0001. ***p < 0.001 Sidak's multiple comparisons. Mean ± SEM and individual mice shown. N = 7 Wt, 8 iKD mice. I) Number of open arm entries in the EPM. T-test, ns. Mean ± SEM and individual mice shown. N = 7 Wt, 8 iKD mice. J) Number of closed arm entries in the EPM. T-test, ns. Mean ± SEM and individual mice shown. N = 7 Wt, 8 iKD mice. K) Schematic of treatments and behavioral testing. Mice received 5d TAM then 3 days later were tested on a hippocampal dependent Y maze, hippocampal dependent object location task (OTL) and an elevated plus maze (EPM). L) Number of mice who chose the novel arm first for entry in Y maze. Fisher's exact test, ns. M) Latency of mice to enter novel arm in Y maze. T-test, ns. Mean ± SEM and individual mice shown. N) Percent time in novel arm over total time in novel and familiar arm. T-test, ns. Mean ± SEM and individual mice shown. O) Percent time spent investigating a moved object in the OLT during training and testing. 2-way ANOVA trial x genotype interaction p = 0.0299. *p = 0.0243 Sidak's multiple comparisons. Mean ± SEM and individual mice shown. P) Total object investigation time in the object location training and testing sessions. 2-way ANOVA trial p < 0.0001. **,***p < 0.01, 0.001 Sidak's multiple comparisons. Mean ± SEM and individual mice shown. Q) Number of open arm entries in the EPM. *p = 0.0493, T-test. Mean ± SEM and individual mice shown. R) Time spent in open arms of the EPM. T-test, ns. Mean ± SEM and individual mice shown. S) Number of closed arm entries in the EPM. T-test, ns. Mean ± SEM and individual mice shown. L-S) N = 27 Wt, 39 iKD mice.

bioRxiv preprint doi: https://doi.org/10.1101/2023.04.24.537801; this version posted April 24, 2023. The copyright holder for this preprint (which was not certified by peer review) is the author/funder, who has granted bioRxiv a license to display the preprint in perpetuity. It is made available under aCC-BY 4.0 International license.

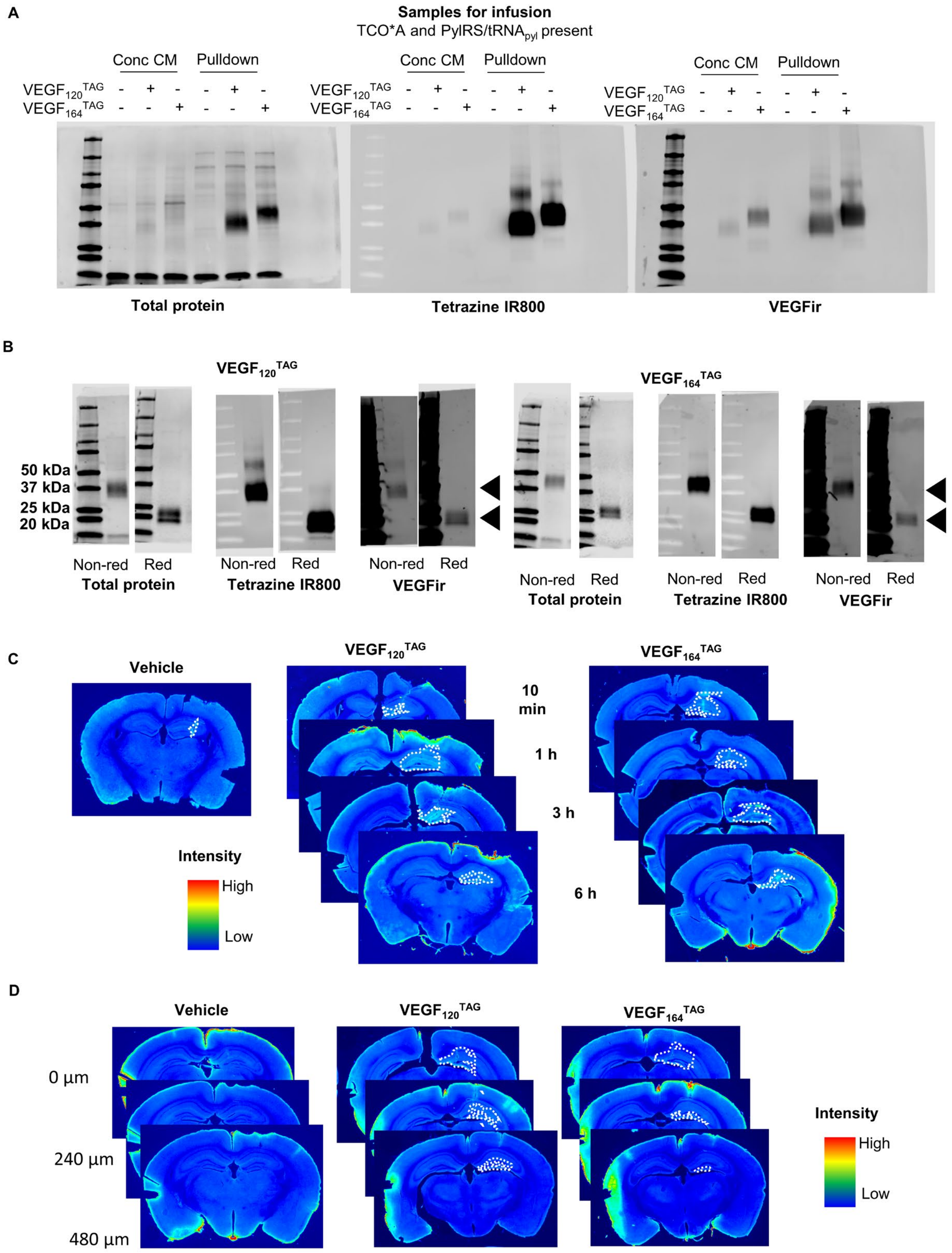


**Supplemental Figure 2. Generation and isolation of VEGF$^{TAG}$ proteins.** A) Images of western blots showing total protein, TCO*A bearing protein (reacted with tetrazine-biotin then streptavidin IR800), and VEGF immunoreactivity (ir) in whole concentrated CM and bead-

bioRxiv preprint doi: https://doi.org/10.1101/2023.04.24.537801; this version posted April 24, 2023. The copyright holder for this preprint (which was not certified by peer review) is the author/funder, who has granted bioRxiv a license to display the preprint in perpetuity. It is made available under aCC-BY 4.0 International license.

concentrated final samples used for infusion. Non-reducing conditions were used, resulting in detection of VEGF dimers. B) Images of western blots run in non-reducing conditions (to reveal VEGF monomers) and reducing conditions (to reveal VEGF dimers). Arrowheads signal dimers and monomers. Blots were replicated at least twice to verify protein identity and tetrazine-reactivity. C) Representative images of VEGF$^{TAG}$ areas over time. Dashed outline shows VEGF$^{TAG}$+ area. Vehicle mouse, VEGF$_{120}^{TAG}$ (3h) mouse and VEGF$_{164}^{TAG}$ (1h) mouse are duplicated from main figure. D) Representative images of VEGF$^{TAG}$ areas in single mice from epicenter to 480 µm rostral. Dashed outline shows VEGF$^{TAG}$+ area.

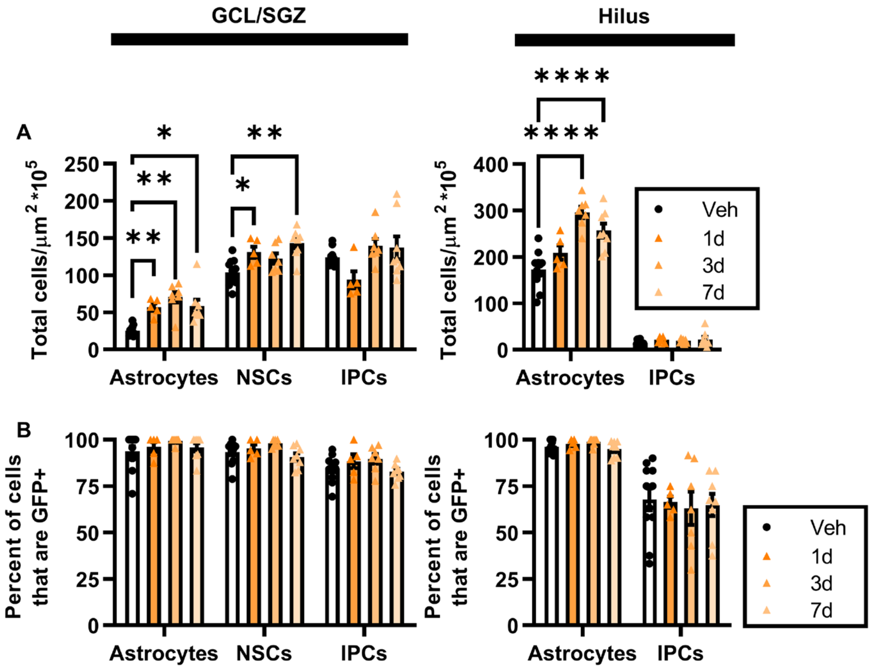


**Supplemental Figure 3. Cell proliferation response in VEGF-GFP mice after KA.** A) Density of total cells in the granule cell layer/subgranular zone (GCL/SGZ) and hilus identified as astrocytes, NSCs or IPCs based on immunolabeling for Sox2 and GFAP quantified 1d, 3d, or 7d after a single systemic injection of KA in adult VEGF-GFP mice. 2 way repeated measures ANOVA: SGZ/GCL: cell type x timepoint $p = 0.0027$, cell type $p < 0.0001$, time $p < 0.0001$. Hilus: cell type x timepoint $p < 0.0001$, cell type $p < 0.0001$, time $p < 0.0001$. *,**,**** $p < 0.05$, 0.01, 0.0001 Dunnett's multiple comparisons to Vehicle. B) Percent of astrocytes, NSCs and IPCs in the granule cell layer/subgranular zone (GCL/SGZ) and hilus identified that were GFP+ 1d, 3d, or 7d after a single systemic injection of KA in adult VEGF-GFP mice. 2 way repeated measures ANOVA: SGZ/GCL: cell type $p < 0.0001$. Hilus: cell type $p < 0.0001$. Dunnett's multiple comparisons to Vehicle all ns. A,B) Mean ± SEM and individual mice shown. N = 11 vehicle, N = 5-8/KA timepoint.

bioRxiv preprint doi: https://doi.org/10.1101/2023.04.24.537801; this version posted April 24, 2023. The copyright holder for this preprint (which was not certified by peer review) is the author/funder, who has granted bioRxiv a license to display the preprint in perpetuity. It is made available under aCC-BY 4.0 International license.

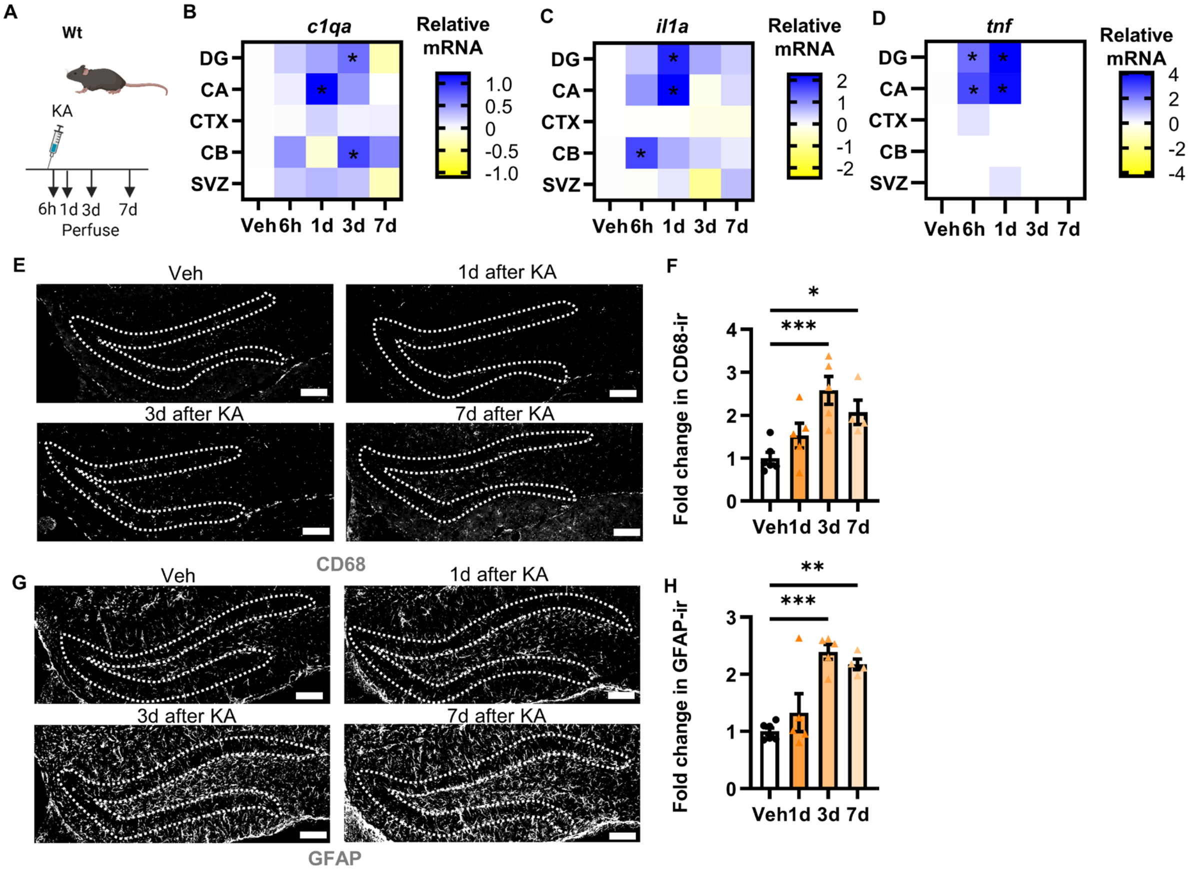


**Supplemental Figure 4. Kainic acid causes DG neuroinflammation.** A) Schematic of treatments. Wt mice received a single injection of systemic KA then were perfused 6h-7d later. B-D) Relative *C1qa (B), Il1a (C)* or *Tnf (D)* mRNA levels in dentate gyrus (DG), CA regions of hippocampus (CA), cortex (CTX), cerebellum (CB) and subventricular zone (SVZ) after KA injection determined by real time qPCR. Two-way ANOVA: C1qa area x treatment interaction $p = 0.0009$, treatment $p < 0.0001$. Il1a area x treatment interaction $p = 0.0005$, treatment $p < 0.0001$, area $p < 0.0001$. Tnf area x treatment interaction $p < 0.0001$, treatment $p < 0.0001$, area $p < 0.0001$. *$p < 0.05$ Dunnett's multiple comparisons versus vehicle within area. N = 20-23 Veh mice, 6-12 KA treated mice/area/timepoint. E) Representative image of CD68 immunoreactivity (ir) in the DG of Wt mice 1d after KA injection. Scale = 100 µm. F) Fold change in CD68 immunoreactivity (ir) thresholded area in the DG of Wt mice 1-7d after KA relative to Veh mice. ANOVA $p = 0.0023$, *,***$p < 0.05$, 0.001 Dunnett's multiple comparisons to Veh. Mean ± SEM and individual mice shown. N = 4-6 mice/group. G) Representative image of GFAP immunoreactivity (ir) in the DG of Wt mice 1-7d after KA injection. Scale = 100 µm. H) Fold change in GFAP immunoreactivity (ir) thresholded area in the DG of Wt mice 1-7d after KA relative to Veh treated mice. ANOVA $p = 0.0001$, **,***$p < 0.01$, 0.001 Dunnett's multiple comparisons to Veh. Mean ± SEM and individual mice shown. N = 4-6 mice/group.

bioRxiv preprint doi: https://doi.org/10.1101/2023.04.24.537801; this version posted April 24, 2023. The copyright holder for this preprint (which was not certified by peer review) is the author/funder, who has granted bioRxiv a license to display the preprint in perpetuity. It is made available under aCC-BY 4.0 International license.

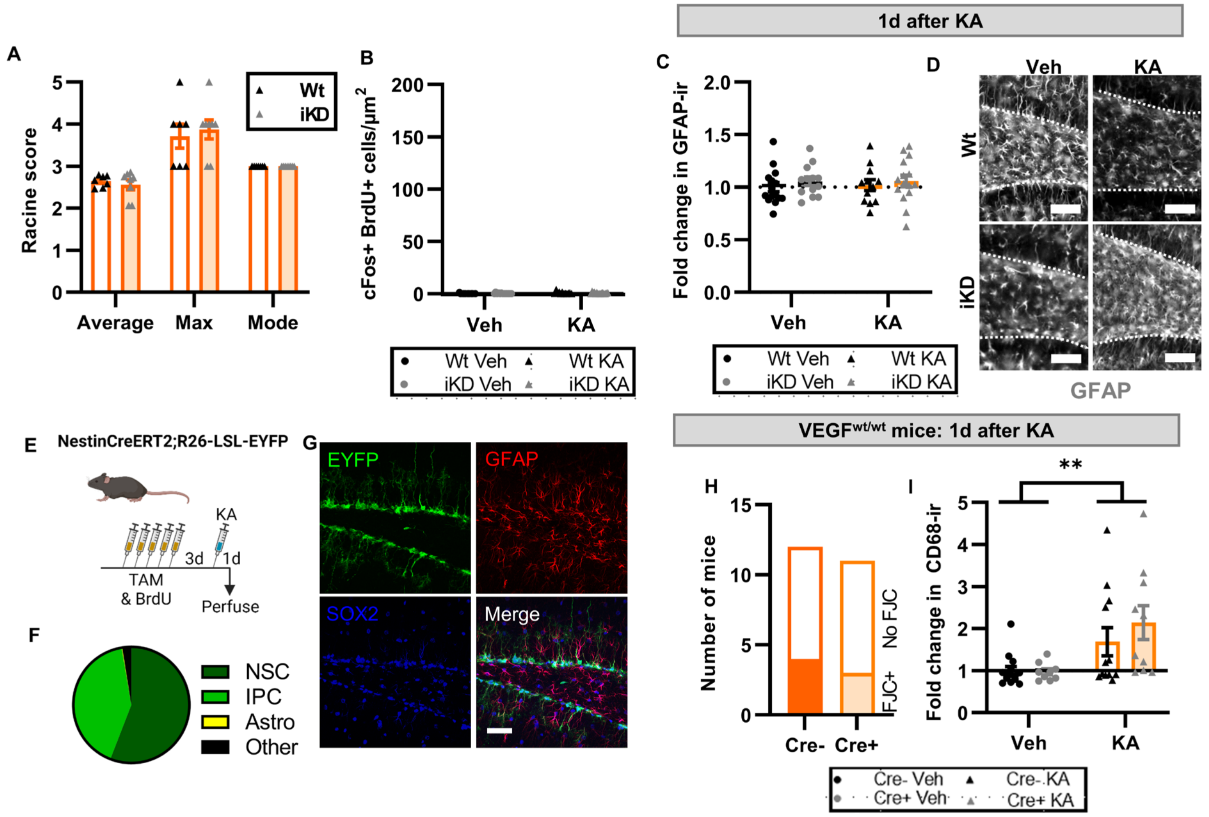


**Supplemental Figure 5. NSPC-VEGF knockdown exacerbates excitotoxic injury.** A) Average, maximum and mode seizure score of Wt and iKD mice according to the Racine scale in the 4h following KA injection. T-tests within each category all ns. Mean ± SEM and individual mice shown. N = 7-8 mice/genotype. B) Density of BrdU+cFos+ cells in the granule cell layer 1d after KA injection in Wt and iKD mice. 2 way ANOVA treatment $p < 0.0001$. C) Fold change in GFAP immunoreactivity (ir) thresholded area in the DG of Wt and iKD mice 1d after KA relative to Wt-Veh mice. 2 way ANOVA all ns. Mean ± SEM and individual mice shown. N = 12-15 mice/group. D) Representative images of GFAPir in the DG of Wt and iKD mice. Scale = 20 µm. E) Schematic of treatments. NestinCreER$^{T2+/-}$;LoxP-STOP-LoxP-EYFP$^{+/-}$ mice received 5d TAM injections followed by a single KA injection 3d after the last TAM injection. Mice were perfused 1d after KA. F) Pie graph showing percent of EYFP+ cells in the DG that where phenotypic NSCs, IPCs, astrocytes or other 1d after KA. N = 3 mice. G) Representative image of EYFP col-labeling with Sox2 and GFAP used to identify astrocytes, NSCs and IPCs. Scale = 20 µm. H) Number of NestinCreER$^{T2-/-}$ and NestinCreER$^{T2+/-}$ mice showing any FJC labeling in the DG 1 d after KA. Fisher's exact test ns. I) Fold change in CD68 immunoreactivity (ir) thresholded area in the DG of NestinCreER$^{T2-/-}$ and NestinCreER$^{T2+/-}$ mice 1d after KA relative to NestinCreER$^{T2-/-}$ Veh treated mice. 2 way ANOVA treatment $p = 0.0011$. Mean ± SEM and individual mice shown. N = 9-14 mice/group.
